# DT-HRL: Mastering Long-Sequence Manipulation with Reimagined Hierarchical Reinforcement Learning

**DOI:** 10.3390/biomimetics10090577

**Published:** 2025-09-01

**Authors:** Junyang Zhang, Yilin Zhang, Honglin Sun, Yifei Zhang, Kenji Hashimoto

**Affiliations:** Graduate School of Information, Production and Systems, Waseda University, Kitakyushu 808-0135, Japan; junyang.z@akane.waseda.jp (J.Z.); zhangyilin@moegi.waseda.jp (Y.Z.); hsun@akane.waseda.jp (H.S.); yifei.zhang@toki.waseda.jp (Y.Z.)

**Keywords:** hierarchical reinforcement learning, long-sequence tasks, decision transformer, robotic manipulation

## Abstract

Robotic manipulators in warehousing and logistics often face complex tasks that involve multiple steps, frequent task switching, and long-term dependencies. Inspired by the hierarchical structure of human motor control, this paper proposes a Hierarchical Reinforcement Learning (HRL) framework utilizing a multi-task goal-conditioned Decision Transformer (MTGC-DT). The high-level policy treats the Markov decision process as a sequence modeling task, allowing the agent to manage temporal dependencies. The low-level policy is made up of parameterized action primitives that handle physical execution. This design improves long-term reasoning and generalization. This method is evaluated on two common logistics manipulation tasks: sequential stacking and spatial sorting with sparse reward and low-quality dataset. The main contributions include introducing a HRL framework that integrates Decision Transformer (DT) with task and goal embeddings, along with a path-efficiency loss (PEL) correction and designing a parameterized, learnable primitive skill library for low-level control to enhance generalization and reusability. Experimental results demonstrate that the proposed Decision Transformer-based Hierarchical Reinforcement Learning (DT-HRL) achieves over a 10% higher success rate and over 8% average reward compared with the baseline, and a normalized score increase of over 2% in the ablation experiments.

## 1. Introduction

In modern warehousing and logistics scenarios, robots are being increasingly deployed [1]. However, they are facing challenges in handling long-sequence complex tasks and multiple tasks. Although conventional deep reinforcement learning (DRL) methods have been widely applied in robotic control, they still struggle with tasks requiring long-term temporal dependencies, broad generalization across environments, and flexible task switching [2,3,4]. To address these challenges, this paper draws inspiration from the hierarchical control architecture of the human nervous system. Specifically, the cortical–basal ganglia–spinal cord pathway of human being provides a multi-layered control paradigm that spans from abstract planning to concrete action execution. Cognitive neuroscience studies have also observed parallels between HRL algorithms and the brain’s cognitive control processes [5,6]. This insight motivated us to build an HRL framework where a high-level policy could capture long-term dependencies and focus on abstract task planning, while a low-level policy would execute fundamental skills that generalize across diverse contexts. Motivated by these insights, this paper introduces a Decision Transformer-based HRL framework designed to efficiently handle multi-task and long-sequence manipulation scenarios.

Generally, HRL employs a high-level and low-level policy to decompose complex tasks into some sub-goals, guiding exploration more effectively and helps alleviate the optimization difficulties caused by long time horizons [7,8]. However, traditional HRL methods often require manually designed sub-goals or carefully crafted reward functions for the high-level policy, and they may perform poorly in tasks with strict sequential constraints. When dense reward feedback is absent, the high-level policy can struggle to learn a reliable strategy for sub-goals setting [9,10].

To overcome these difficulties, this paper proposes a hierarchical RL framework with the high-level policy based on an improved Decision Transformer. The Decision Transformer is a recent approach that reframes reinforcement learning as a sequence modeling problem, using an autoregressive Transformer model to directly learn a mapping from sequences of states, actions, and returns-to-go to the agent’s policy [11]. Unlike traditional RL methods that optimize value functions or policy networks, the Decision Transformer leverages the powerful sequence modeling capabilities of the Transformer architecture to capture long-range dependencies from diverse offline trajectories. Moreover, as it essentially follows a deep learning paradigm without explicit exploration, it inherently avoids the instability often introduced by the exploration process in reinforcement learning. This paper incorporates the Decision Transformer into the high-level policy of the HRL architecture, replacing the conventional subgoal-selection mechanism. This eliminates the need for complex subgoal reward engineering and allows the agent to directly learn optimal action sequences for completing tasks from offline demonstration data.

However, relying on the standard Decision Transformer alone is insufficient for handling diverse task scenarios. Its use of future accumulate reward, return-to-go (RTG), as the sole conditioning signal does not fully align with the demands of multi-step robotic manipulation tasks, where task semantics and goal configurations may vary significantly [12,13,14]. To address this limitation, a task embedding and goal embedding is introduced as additional conditioning inputs. These embeddings provide explicit guidance about the current task and target state, enabling the high-level model to adapt its policy accordingly. This conditional design allows the model to dynamically adjust its internal strategy when switching between tasks. In addition, replacing the return-to-go with a goal embedding may impair the model’s ability to distinguish between sub-optimal and shortest-path solutions, as trajectory quality is no longer explicitly encoded. To mitigate this issue, this paper augments the sequence modeling loss with a path-efficiency regularization term, which encourages the model to prefer shorter, more direct-action sequences toward the goal. This adjustment compensates for the loss of explicit reward-to-go signals, while preserving trajectory optimality in sparse-reward, multi-step tasks. As a result, a single model can handle multiple tasks without sacrificing generalization ability.

At the same time, this paper implements the low-level policy as a library of parameterized action primitives. These primitives are basic skills that are either predefined or learned offline, and each can directly execute a basic motion. The high-level Decision Transformer outputs discrete macro-action commands, which the low-level controller interprets and executes using the appropriate primitives. This restricts the high-level’s exploration to a finite set of semantic skills, rather than a continuous action space, greatly improving learning efficiency. Moreover, because the low-level primitives are task-agnostic and reusable, the framework can easily adapt to new tasks [15,16].

In summary, the challenges addressed in this study can be distilled into the following research gaps. First, existing HRL approaches are often unable to effectively capture long-term temporal dependencies, which may cause the learned policies to become short-sighted in practical tasks. Second, reinforcement learning methods typically require carefully engineered reward functions, which limit their practical applicability. Third, current methods lack sufficient generalization ability across diverse tasks and goals, restricting their deployment.

The objective of this study is to propose a HRL framework utilizing an MTGC-DT module and a library of action primitives to effectively handle long-sequence robotic manipulation tasks, improving long-term reasoning, task generalization in sparse-reward and noisy data scenarios common in warehousing and logistics applications. The main contributions of this work are as follows:

- Proposing a hierarchical reinforcement learning framework that integrates the Decision Transformer into the high-level decision-making process. By utilizing tasks and goal embeddings, the model can handle multiple tasks and target configurations using a single high-level controller. In addition, a path-efficiency loss correction is introduced after return-to-go prompt is deprecated, preventing the agent from falling into local optimality.
- Proposing a parameterized and learnable action primitive library for the low-level controller. This paper constructs a modular set of motor skills, allowing the low-level policy to generalize across different tasks. These primitives are shared across tasks to improve reusability and adaptability.
- Validating the proposed method on distinct long-sequence manipulation tasks. For each task, this paper collected over ten thousand offline trajectories with mixed quality to form training datasets. Comparative results show that DT-HRL significantly outperforms traditional HRL baselines in terms of success rate and final reward, particularly under sparse reward settings and noisy offline data.

The remainder of this paper is organized as follows: Section 2 reviews related work, including the use of sequence modeling in RL and prior studies on long-sequence multi-task control. Section 3 covers the necessary preliminaries, including hierarchical policy frameworks, the Proximal Policy Optimization (PPO) algorithm, and causal Transformer. Section 4 details the proposed approach, including the problem formulation, the overall DT-HRL architecture, and the design of the high-level Decision Transformer and low-level action primitives. Section 5 presents the experimental setup and results, with comparative evaluations against baseline methods. Section 6 summarizes the overall work and highlights the main contributions of this study. It further discusses the limitations of the proposed approach and explores potential directions for future research.

## 2. Related Work

### 2.1. Multi-Task and Long-Sequence Robot Manipulation

Multi-task and long-sequence manipulation refers to robotic control scenarios where the system must accomplish a variety of tasks, each involving a sequence of temporally extended actions. In multi-task settings, the robot operates across distinct goal conditions, object configurations, or task specifications. Long-sequence manipulation involves tasks that must be decomposed into multiple intermediate stages or subgoals, where later actions depend on the successful execution of earlier steps. These problems typically exhibit sparse or delayed rewards, complex contact interactions, and high variability in temporal structure, making them challenging for planning, generalization, and policy evaluation [17,18,19,20].

In scenarios where robotic arms must perform multi-task and long-sequence manipulations, conventional approaches include policy gradient methods with reward shaping and HRL with subtask scheduling. In multi-task learning, typical studies aim to train a general policy capable of handling diverse tasks. These approaches often rely on shared state representations and policy structures but may suffer from performance degradation when switching between tasks [21]. For long-sequence tasks, strategies such as reward shaping and guided sub-goals have been proposed to address the sparse reward problem. However, these methods often depend heavily on domain-specific knowledge and lack general applicability [22]. Some research has explored learning from demonstrations, where human or scripted expert trajectories are used to guide policy learning. While this can alleviate issues related to sparse rewards, demonstration data in multi-task, multi-step settings tend to be large and complex, making it challenging to train a generalizable policy efficiently. This work introduces sequence modeling to address this problem, leveraging Transformer to model diverse multi-task demonstration trajectories, aiming to tackle both task generalization and long-sequence planning.

### 2.2. Hierarchical Reinforcement Learning Method

Hierarchical reinforcement learning has been widely explored as a solution to long-sequence decision-making problems in robot manipulation. Classic frameworks such as Hierarchical Actor-Critic (HAC) and Data-Efficient Hierarchical Reinforcement Learning (HIRO) adopt two-level architecture, where the high-level policy periodically outputs subgoals or abstract actions, and the low-level policy learns to achieve them over shorter time spans [7,23]. These subgoals are often specified in state space or formulated as parameterized skills. This structure helps improve exploration efficiency and mitigate credit assignment issues over long-time horizons.

Despite these advantages, many HRL approaches rely on carefully designed high-level policies and auxiliary objectives. For example, HAC requires learning a high-level value function to assess the utility of subgoals, which can be difficult to train under sparse reward conditions. Some methods introduce affordance feedback mechanisms to estimate the rationality of subgoals settings, penalizing unachievable subgoals generated by the high-level policy. But this increases algorithmic complexity and tuning effort. In addition, multi-task scenarios introduce further challenges, as separate high-level policies are often required for different task families [10].

To address these limitations, recent work incorporates a library of behavior primitives or motion skills, enabling the high-level policy to operate over a set of pre-trained and reusable action primitives [24,25,26,27]. In this context, the high-level policy does not need to specify subgoals explicitly, but instead sequences skill primitives to complete the overall task. Our method further integrates Decision Transformer to model successful behavior sequences over primitives, alleviating the need for hand-designed high-level rewards or credit assignment structures.

### 2.3. Sequence Modeling in Reinforcement Learning

Reformulating decision-making problems of traditional reinforcement learning frameworks into sequence modeling is a new idea that has emerged in recent years. A representative example is Decision Transformer [11], which models trajectories as sequences of return-to-go, states, and actions. Instead of relying on value functions or policy gradients, DT uses a causal Transformer to predict actions conditioned on past experience and target returns. This formulation allows the model to directly leverage advances in sequence modeling, while avoiding issues such as temporal difference bootstrapping and instability in value estimation.

While DT has shown competitive performance in offline reinforcement learning tasks such as Atari and continuous control benchmarks, its direct application to robotic manipulation remains limited. One challenge is that robotic tasks often lack a well-defined or easily computable target return, making it difficult to construct return-based prompts as used in the original DT framework [28]. Moreover, robotic environments typically involve long-sequence planning with structured subgoals and physical constraints, which differs from the settings in which DT has been primarily evaluated. In addition, the original DT architecture is not inherently designed to handle multiple heterogeneous tasks within a single model, which limits its scalability in multi-task manipulation scenarios [29].

To address these limitations, recent work has proposed integrating hierarchical structures into sequence modeling frameworks. For instance, André Correia et al. introduced Hierarchical Decision Transformer (HDT) to organize the transformer layers into levels corresponding to different temporal resolutions, aiming to improve long-sequence credit assignment [12]. In parallel, Kong et al. introduced Mixture-of-Experts Decision Transformer, which extends DT with a mixture-of-experts mechanism to improve scalability and generalization across diverse task scenarios [13]. Nevertheless, the application of sequence modeling methods to multi-task and long-sequence robotic control remains an open research direction. Existing approaches often assume access to expert demonstrations or structured action representations, and their effectiveness under sparse reward or limited data conditions has yet to be fully validated.

## 3. Preliminaries

### 3.1. Hierarchical Reinforcement Learning Framework

HRL aims to overcome the challenges of complex, long-sequence decision-making tasks by organizing the learning problem into multiple levels of abstraction. Instead of solving the problem using a single, flat policy, HRL introduces multiple decision-making layers, each operating at different temporal and spatial granularities [8,30].

**Figure 1:**
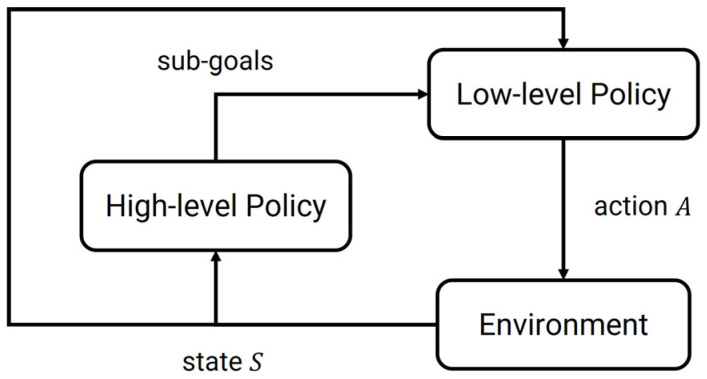
Hierarchical Reinforcement Learning.

As shown in Figure 1, typically, an HRL framework consists of two distinct types of policies:

- High-level policy $\left(\pi_{H}\right)$: $\pi_{H}$ is responsible for selecting abstract actions or goals at a coarser timescale, which is represented as:
  (1) $$\pi_{H}\left(g_{t}|s_{t}\right)$$

- Low-level policy $\left(\pi_{L}\right)$: $\pi_{L}$ is responsible for executing the selected abstract actions using concrete primitive actions, which is represented as:
  (2) $$\pi_{L}\left(a_{t}|s_{t},g_{t}\right).$$

Low-level policy selects primitive actions $a_{t}$ conditioned on the current state $s_{t}$ and high-level instruction $g_{t}$. The high-level policy typically operates on a reduced or abstract state space, while the low-level policy operates on the original state space.

By abstracting tasks into higher-level goals, HRL can guide exploration more efficiently. Decomposition into hierarchical structures also helps mitigate the complexity arising from long-sequence decision problems. In addition, learned lower-level skills can often be reused across tasks, enabling better generalization.

In this paper, an HRL framework is adopted to efficiently handle complex multi-step sequential decision-making tasks. The specific algorithms and their implementations will be discussed in subsequent sections.

### 3.2. Proximal Policy Optimization Algorithm

The Actor-Critic method is DRL algorithm which integrates both policy-based and value-based methods. In this framework, the policy, known as the actor, explicitly parameterizes the agent’s decision-making strategy, denoted as $\pi_{\theta }\left(a|s\right)$, guiding the agent’s action selection. Concurrently, the value function, known as the critic, approximates the expected return or advantage associated with states or state–action pairs, represented as $V_{\phi }\left(s\right)$ or $Q_{\psi }\left(s,a\right)$, thus providing a learned baseline for policy improvement. The actor is updated by maximizing expected returns guided by gradients computed from critic-provided advantage estimates, typically formulated as:

(3) $$\nabla_{\theta }J\left(\theta \right)=E_{\pi_{\theta }}\left[\nabla_{\theta }\log\pi_{\theta }\left(a_{t}|s_{t}\right)\;A_{t}\right]$$

where $A_{t}$ represents the advantage signal, indicating how much better or worse an action is compared to the average expectation. The critic, on the other hand, updates its parameters by minimizing prediction errors on returns or value targets derived from experience data [31,32].

Prominent algorithms in this category include Asynchronous Advantage Actor-Critic (A3C) and Proximal Policy Optimization. These model-free DRL methods have been successfully applied to continuous-control tasks in robotics and simulated locomotion, demonstrating that end-to-end learning with minimal handcrafting can solve complex control problems.

A primary advantage of actor-critic methods is their ability to simultaneously leverage explicit policy optimization for stable convergence, along with value-based estimations to reduce variance, significantly improving sample efficiency and training stability in complex, high-dimensional decision-making tasks. Among them, Proximal Policy Optimization algorithm is a state-of-the-art method known for its stability and efficiency. The core of the PPO algorithm is to constrain the optimization process while optimizing the objective function so that each strategy update will not deviate too far from the original strategy, thereby ensuring the stability and convergence of the training process [2,33]. The objective function can be expressed as follows:

(4) $$L^{\mathrm{CLIP}}\left(\theta \right)=E_{t}\left[\min\left(\frac{\pi_{\theta }\left(a_{t}|s_{t}\right)}{\pi_{\theta_{\mathrm{old}}}\left(a_{t}|s_{t}\right)}A_{t},\right.\right.\left.\left.\mathrm{clip}\left(\frac{\pi_{\theta }\left(a_{t}|s_{t}\right)}{\pi_{\theta_{\mathrm{old}}}\left(a_{t}|s_{t}\right)},1-\epsilon ,1+\epsilon \right)A_{t}\right)\right]$$

where $\pi_{\theta }$ is the current policy, $\pi_{\theta_{\mathrm{old}}}$ is the old policy, $A_{t}$ is the advantage function, ϵ is the clipping threshold.

The algorithm employs an Actor network to approximate the behavior policy and a Critic network to approximate the advantage function. The Actor outputs an action $a_{t}$ based on the current state $s_{t}$, and the environment transitions to the next state. The Critic evaluates the action taken by the Actor by estimating the advantage function $A_{t}$, which measures how much better or worse the chosen action is compared to the average expected return from state $s_{t}$. A clipping function is used to limit the difference between the new and old policies. This clipping function calculates the ratio of the new policy to the old policy for each action sample and compares it to a predefined range [34].

In this paper, the PPO algorithm is used for exploring actions and selecting parameters in the low-level network. The specific implementations will be discussed in subsequent sections.

### 3.3. Causal Transformer and Autoregressive Sequence Modeling

The Causal Transformer has emerged as a prominent neural network architecture in recent years, particularly within the fields of Natural Language Processing (NLP) and time series analysis. By introducing a causal constraint to the standard Transformer model, it is adept at capturing the sequential order and temporal dependency tasks [35].

The standard Transformer architecture employs a self-attention mechanism that permits each element in a sequence to attend to all other elements, irrespective of their relative positions. While this bidirectional attention proves effective for tasks such as sequence-to-sequence encoding, it is unsuitable for scenarios where causality must be preserved. In many applications such as text generation, the prediction of a future state must not be influenced by information from that future state itself.

The Causal Transformer is designed to address this limitation. It operates under the core principle of constraining the model to attend only to information from previous positions at any given time step. All information from future positions is masked, thereby enforcing a strict and unidirectional flow of information that defines the model’s autoregressive property.

This causal constraint is implemented through a modification to the self-attention mechanism, which is also known as masked self-attention. Subsequent to the standard calculation of attention scores between queries (Q) and keys (K), a mask matrix (M) is applied. This mask is typically an upper-triangular matrix where elements corresponding to future positions are set to a large negative value, while all other elements are zero. This mask is added to the scaled dot-product of the queries and keys before the SoftMax operation, as shown in the equation:

(5) $$\mathrm{Causal}\;\mathrm{Attention}\left(Q,K,V\right)=\mathrm{softmax}\left(\frac{QK^{T}}{\sqrt{d_{k}}}+M\right)V$$

where *M* is the mask matrix:

(6) $$M_{ij}=\left\{\begin{array}{ll} 0 & \mathrm{if}\;i\geq j\\ -\infty & \mathrm{if}\;i<j \end{array}\right..$$

The application of this mask ensures that the attention weights for any position i are distributed exclusively among the current and preceding positions (j≤i), effectively preventing any flow of information from the future and preserving the causal structure of the sequence [36].

In this paper, Causal Transformer is used for sequence modeling for the reinforcement learning trajectories. The specific implementations will be discussed in subsequent sections.

## 4. Methodology

### 4.1. Problem Formulation

This research addresses the challenge of enabling a robotic agent to solve long-sequence, multi-task manipulation problems that require complex reasoning and sequential decision-making. As outlined in the Introduction, the proposed hierarchical approach relies on action primitives that are not time-constant but vary according to the number of time steps of the low-level actions. To accurately capture these temporally extended actions, this paper models the high-level decision-making process as a Semi-Markov Decision Process (SMDP) [37].

An SMDP is defined by the tuple (S, O, P, R, γ), where the components are instantiated for the robotic tasks as follows:

- State Space (*S*): *S* is the high-level, abstract observation space. Each state $s_{t}\in S$ provides the necessary information for strategic decision-making. It is a fixed-dimension vector composed of environmental information, the agent’s internal state and the goal condition.
- Option Space (*O*): *O* is a discrete set of high-level options, or macro-actions, that the agent can choose. An option o∈O corresponds to a temporally extended task with clear meaning. The agent’s policy operates by selecting a sequence of these options.
- Transition Probability (*P*): The function P(s’,τ∣s,o) defines the probability of transitioning to a new high-level state s’ after executing option o from state s, where τ is a random variable representing the number of timesteps the option took to complete.
- Reward Function (*R*): R(s,o) represents the total reward accumulated from the environment during the entire execution of option o after it was initiated in state s.

The objective is to learn an optimal high-level policy, denoted as $\pi_{H}:S\to O$, which maps a given state to the best option to execute. The policy is optimized to maximize the expected cumulative discounted reward over an episode. The objective function $J(\pi_{H})$ is defined as:

(7) $$J\left(\pi_{H}\right)=E_{\pi_{H}}\left[\sum_{k=0}^{\infty }\gamma^{T_{k}}R_{k+1}\right]$$

where k indexes the sequence of high-level decisions. $R_{k+1}$ is the cumulative reward received after completing the option. γ∈[0, 1] is the discount factor. The term $T^{k}$ represents the cumulative time elapsed before the k-th decision is made, which accounts for the variable duration of each option. It is defined as:

(8) $$T_{k}=\sum_{i=0}^{k-1}\tau_{i}$$

where $\tau_{i}$ is the duration of the i-th option. This formulation represents the discounting of rewards over variable time intervals, which is central to the SMDP framework.

Having established this formal framework, the following sections will introduce the proposed DT-HRL architecture, which is designed to learn the optimal policy $\pi_{H}$ for this SMDP.

### 4.2. DT-HRL Framework

**Figure 2:**
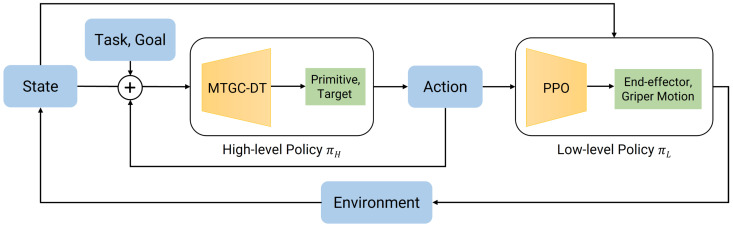
DT-HRL Pipeline. Blue blocks denote inputs and outputs, yellow blocks represent policy modules, and green blocks indicate intermediate targets and control commands.

To solve the SMDP formulated in Section 4.1, this paper proposes an innovative Decision Transformer-based Hierarchical Reinforcement Learning framework. This framework represents a paradigm shift from conventional value-based HRL methods. Instead of learning a complex value function for goal selection, DT-HRL re-contextualizes the hierarchical reinforcement learning problem as a conditional sequence modeling task. The overall architecture, depicted in Figure 2, consists of a two-tiered hierarchy: a high-level policy $\pi_{H}$ realized by an MTGC-DT module; and a low-level policy $\pi_{L}$ implemented as a library of action primitives.

A core innovation of this framework lies in replacing the conventional goal-conditioned reinforcement learning policy at the high level with a Decision Transformer. This architectural choice directly addresses the critical limitations of methods like HAC in tasks with strong sequential dependencies. DT’s causal self-attention mechanism is innately suited to modeling long-term temporal structures within an entire task trajectory. Rather than learning to reach a series of independent sub-goals, the high-level policy learns the contextual, ordered sequence of actions that leads to successful task completion. Furthermore, this approach fundamentally obviates the need for intricate, hand-engineered reward functions. The Decision Transformer reframes the original problem as conditional sequence modeling. By learning the relationships between states, actions, and their ultimate outcomes across a diverse dataset of behaviors, the model acquires a much richer understanding of the task dynamics. This allows it to generate effective, goal-directed strategies without relying on step-by-step reward shaping, thus greatly simplifying the problem.

However, the introduction of the DT method brings a new problem: the model may lose its generalization ability for different tasks. To address this challenge, a clear mechanism to label different tasks is required. In this case, task-specific embeddings are incorporated into the input representation. Specifically, a discretized vector representation of a set of tasks is projected into a high-dimensional embedding vector via a learned hidden layer. This task-specific embedding is then integrated as a global contextual bias to the embedding of every token in the input sequence. This process effectively shifts the entire trajectory representation into a task-specific subspace within the model’s latent space. It allows the subsequent Transformer layers to apply distinct, task-specialized transformations to the input sequence, enabling the monolithic model to dynamically instantiate different behavioral “personalities” appropriate for the given task. Consequently, the model learns not a single-function policy, but a versatile, conditional generator capable of producing proper action sequences for each task in its library.

At the lower tier of the hierarchy, DT-HRL addresses the challenge of inefficient exploration by employing a library of meaningful, task-agnostic action primitives for $\pi_{L}$. Instead of having the high-level policy explore a high-dimensional, continuous control space to discover sub-goals, it operates within a constrained, discrete action space of semantic skills. This structured action space drastically prunes the exploration problem, enabling more efficient and targeted learning of the high-level strategy. The details of this framework are shown as Algorithm 1.
**Algorithm 1** Decision Transformer–based Hierarchical Reinforcement Learning (DT-HRL)**Input:** Offline dataset 𝒟, action primitive set 𝒫, task embedding
$\phi_{\mathrm{task}}\left(\cdot \right)$, goal embedding $\phi_{\mathrm{goal}}\left(\cdot \right)$, learning rate η, path-efficiency loss term $L_{\mathrm{PE}}$
**Output:** Hierarchical policy
$\pi =\{\;\pi_{\theta }^{high},\pi_{\theta }^{low}\;\}$
**// Train High-Level Decision Transformer //**
1.  Initialization

2.  **for** episode **do**

3.       Sample a task τ and trajectory
$\;\{{\left(s_{t},a_{t},r_{t},g_{t}\right)}_{t=0}^{T}$ from 𝒟

4.      
$S=\left[s_{t-H},\ldots ,s_{t}\right],\;\;A=\left[a_{t-H},\ldots ,a_{t}\right]$

5.      $\;{â}_{t+1}\leftarrow \pi_{\theta }^{high}\left(S,A,\phi_{task}\left(\tau \right),\phi_{goal}\left(g_{t}\right)\right)$

6.     
$\;a_{H}={â}_{t+1}$             ▷ Delivered to low-level policy

7.     
$\;L\leftarrow \mathrm{CrossEntropy}\left({â}_{t+1},a_{t+1}\right)+\lambda L_{PE}$

8.      
$\theta \leftarrow \theta -\eta \nabla_{\theta }L$

9.  **end for**

**// Low-Level Execution //**
10.  function $\pi_{\theta }^{low}\left(a_{H}\right)$

11.      
$s_{0}\leftarrow \mathrm{initial}\mathrm{state}$

12.       **for**
 k = 0 to K **do**

13.            
$\;a_{k+1}\leftarrow \pi_{\theta }^{low}\left(s_{k},a_{H}\right)$       ▷ Execute low-level controller

14.            
$\;s_{k+1}\leftarrow Environment\left(s_{k},a_{k+1}\right)$

15.              **if** done **then**

16.                   **break**

17.              **end if**

18.        **end for**

19.  **end function**


In summary, the DT-HRL framework synergistically combines the powerful sequence modeling capabilities of causal Transformer with the structural efficiencies of hierarchical learning. The key difference between DT-HRL and existing HRL method is shown in Table 1.

### 4.3. High-Level Policy: A Multi-Task Goal-Conditioned Decision Transformer

In any standard reinforcement learning formulation, the interaction between an agent and its environment is fundamentally characterized by a sequence of three key elements: state, action, and return. The paradigm of Decision Transformer departs from traditional methods, which typically learn value functions or policies from these elements. Instead, it reframes the Markov decision problem by explicitly modeling the joint distribution of the entire sequence via an autoregressive Transformer.

In the original Decision Transformer, each token triple contains a return-to-go value, whose magnitude implicitly steers the policy toward high-reward trajectories. This mechanism works well in game-style benchmarks, but is not suitable for complex robotic manipulation: reward signals in such domains are typically difficult to compute or estimate reliably. RTG becomes a coarse, weakly shaped scalar that offers little guidance and forces the model to extrapolate toward an artificial “maximum-RTG” that may not correspond to the shortest or safest motion. Therefore, RTG as a performance prompt is omitted and instead prepend two learned context vectors: a task embedding that disambiguates the skill family and a goal embedding that encodes the desired objective state. Conditioning on an explicit goal is more intuitive for human specification, aligns naturally with manipulation objectives, and provides the transformer with a direct, reward-agnostic target, thereby eliminating the need to predict or normalize RTG while yielding finer control over path efficiency.

However, removing return-to-go conditioning may reduce the model’s sensitivity to the quality of individual trajectories, particularly its ability to distinguish between optimal and sub-optimal solutions. To mitigate this issue, this paper introduces a path-efficiency regularization term into the sequence modeling loss which is defined as:

(9) $$L_{\mathrm{PE}}=1-e^{-\lambda_{\mathrm{PE}}T}$$

where $\lambda_{\mathrm{PE}}$ is the penalty coefficient controlling the growth rate of the path-efficiency loss. This term penalizes unnecessarily long or indirect action sequences, encouraging the model to generate trajectories that reach the goal in fewer steps. Formally, the total training objective becomes a weighted sum of the behavior cloning loss and a path-efficiency cost, computed based on the deviation from a direct path. This adjustment restores a form of trajectory quality supervision in the absence of explicit return signals.

**Figure 3:**
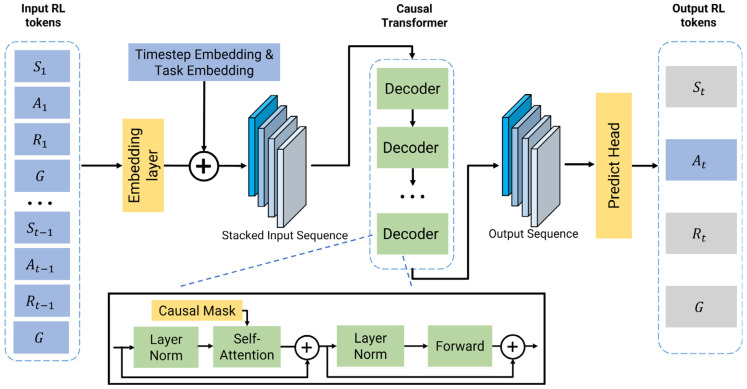
Multi-task goal-conditioned DT structure. Blue blocks denote input and output, yellow blocks represent modules, and green blocks indicate Transformer components. Grey blocks mark elements not directly used. The plus sign represents concatenation or residual addition, dotted boxes denote stacked structures, and the dotted arrow highlights a magnified view of the decoder block.

As shown in Figure 3, the architecture consists of three main components: an embedding layer to tokenize the input trajectory, a stack of causal Transformer blocks for contextual processing, and a predict head for action prediction. For efficiency and flexibility, this paper implements the causal transformer using custom Transformer module, which is significantly lighter-weight compared to pre-trained GPT-2 architectures. This setup allows us to reduce the network size and unnecessary parameter overhead.

The initial step in modeling this joint distribution involves tokenizing each constituent element of a trajectory. For any given time step t, the framework processes three fundamental inputs: the return $R_{t}$, the state $s_{t}$, and the preceding action $a_{t-1}$. These elements are projected into a unified high-dimensional latent space through dedicated embedding layers.

To provide essential temporal context, a learned positional embedding, derived from the absolute timestep t within the episode, is added to each of these token embeddings. Furthermore, to enable a single model to adeptly handle multiple tasks, this paper introduces a crucial contextual modulation step. A discrete task-specific vector is projected into a high-dimensional embedding. This task-specific vector, along with a corresponding goal embedding, serves as a global contextual signal that is added to each of the aforementioned token embeddings. This effectively conditions the entire input sequence on the specific task to be performed, allowing the subsequent Transformer layers to apply task-specialized processing. These fully contextualized embeddings are then arranged into an interleaved sequence for processing.

The sequence of token embeddings is fed into a stack of Transformer encoder layers. A crucial feature of these layers is the application of a causal self-attention mask. This mask ensures that the model’s output for any given time step t can only depend on inputs from the past. This autoregressive property is critical for ensuring that the policy is logically realizable, as it prevents the model from accessing future information when making a decision. Without such mask designing, the self-attention mechanism could attend to the entire sequence acausally. It will create a shortcut that allows the model to “cheat” by accessing the ground-truth action when it is tasked with predicting that very action. Consequently, the model would fail to learn anything meaningful. This causal structure enables the model to learn how to predict an action based on the history of events leading up to that point. The final output embedding for each token is not merely a representation of itself, but a contextually rich vector, making it a substrate for predicting the subsequent action.

To generate an action based on a given state and target return, the model takes the corresponding output embedding from the final Transformer layer, which is passed through a predict head to produce logits over the discrete high-level action space O. An action is then sampled from this distribution. This output sequence is subsequently decoded by the predict head into a semantic pair (primitive, target) for execution by the low-level policy.

### 4.4. Low-Level Policy: State-Conditioned Generic Action Primitives

The DT-HRL framework realizes the decoupling of ‘what to do’ from ‘how to do’ through its two-tiered architecture. Within this structure, the low-level policy serves as the crucial action execution engine, responsible for translating the abstract commands issued by the high-level policy into concrete physical actions in the environment. However, this low-level policy is implemented not as a learned neural network, but as a deterministic library of reusable, parameterized skills known as action primitives. This library includes motion skills with explicit semantics such as grasping, placing, moving, and twisting, which build a general-purpose repertoire of fundamental manipulation skills.

A key architectural principle of this framework is that the low-level actions are not explicitly generated by the high-level policy. This contrasts with many traditional HRL approaches where the high-level policy might output a sub-goal in the state space, which is usually a target coordinate. Instead, the concrete target for a primitive is dynamically computed at runtime, conditioned on both the abstract command from high-level policy and the current physical state of the environment. This process could be illustrated as follows:

- State Query: It queries the physics simulator to obtain the real-time physical states.
- Target Calculation: The primitive takes the state as a parameter and calculates a sequence of waypoints for the robot’s end-effector.
- Execution: These calculated waypoints are then passed to the underlying motion controller for execution.

All primitive actions in the library are parameterized and executed through this unified three-step pipeline. This standardized design ensures that each primitive adapts to the real-time environment state, thereby enabling precise coordination across sequential primitives.

Primitive actions requiring fine-grained adaptation to object positions, are realized through a learned policy. To this end, this paper formulates the task of learning an optimal motion as a reinforcement learning problem, solved using the PPO algorithm. The policy’s state space comprises a concatenation of the end-effector position and the real-time coordinates of the target object, which is provided by the RGB-D camera. The action space is defined as a 3-dimension coordinate increment with the gripper’s motion (open or close). At each step, the policy outputs an incremental adjustment, which approaches the target position. Through this incremental exploration, the policy learns to navigate towards the optimal position. The learning process is guided by a shaped reward function that provides positive feedback for reducing the distance to the target. The final optimized coordinates are passed to the controller to execute the physical action.

Each primitive is a specialized routine adept at handling the control complexities of its specific skill, ensuring reliable execution. More significantly, this approach offers substantial advantages for task generalization. The primitives themselves are inherently task-agnostic. Consequently, extending the agent’s capabilities to novel tasks does not necessitate retraining low-level motor control. This compositional approach allows for rapid adaptation to new objectives and significantly enhances the overall generality and scalability.

## 5. Experiments and Results

This section presents a comprehensive empirical evaluation of the proposed DT-HRL framework. A series of experiments is designed and conducted within a simulated robotic manipulation environment to investigate the performance of our work. Specifically, this evaluation seeks to answer three key research questions:

1. How does this DT-HRL framework compare against conventional HAC baselines in terms of final performance?
2. What is the robustness and adaptability of the proposed approach when trained on offline datasets of low quality and under sparse reward structures?
3. What are the crucial contributions of the task embedding, goal embedding and PEL to the proposed framework?

### 5.1. Experimental Setup

**Figure 4:**
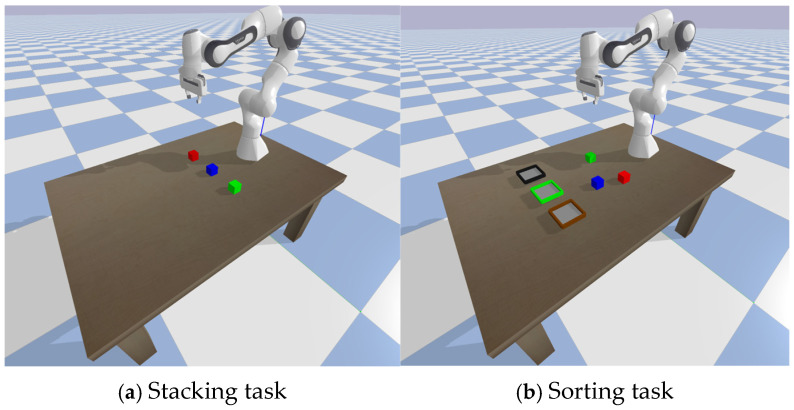
Experiments setting. In the Sorting task, three containers are randomly chosen from Red, Green, Blue (targets) and Brown, Black (distractors), and placed into three spatial slots.

To evaluate the proposed DT-HRL framework, this paper designs two distinct long-sequence manipulation tasks as shown in Figure 4: Sequential Stacking and Spatial Sorting.

Sequential Stacking: the primary objective of this task is to evaluate the policy’s ability to learn and execute plans with strong sequential dependencies. In each episode, three distinct cubes are placed at random initial positions on a tabletop. The agent is provided with a randomly sampled target configuration which specifies the required vertical stacking order. To succeed, the agent must infer and execute the correct sequence of action primitives, respecting the physical constraints. The wrong order will cause the task to be incomplete or get stuck in the middle. This task directly assesses the model’s capacity for long-sequence planning and modeling temporal dependencies.

Spatial Sorting: this task is designed to be more challenging, evaluating the agent’s capacity for dynamic spatial reasoning and generalization in the presence of distractors. The environment setup consists of three fixed spatial slots on the tabletop. In each episode, three containers are randomly selected from a larger pool of five types (Red, Green, Blue, Brown, and Black, with Brown and Black being distractors) and are randomly assigned to these three slots. To succeed, the agent must first identify the identities of the containers at each spatial location from its observation and then form a correct mapping between the cube it is holding and the appropriate spatial slot. The inclusion of non-target distractor containers requires the policy to learn to inhibit actions directed towards irrelevant locations. Success is achieved only if all required cubes are placed in their corresponding-colored containers, provided those containers are present in the current episode. This task probes the model’s ability to perform relational reasoning and execute context-dependent policies.

### 5.2. Training Details

The DT-HRL architecture as shown in Figure 3. is adopted for training. The full set of hyperparameters is summarized in Table 2. All experiments are conducted in Pybullet simulator on a single RTX 4070 GPU (NVIDIA Corporation, Santa Clara, CA, USA).

The discount factor γ is typically chosen within the range 0.95–0.99 in practical applications. The higher values place more emphasis on long-term rewards and lower values focus on short-term returns. Considering the long-sequence nature of these tasks. The discount factor is set as γ=0.99. The reward function R adopts a sparse binary design, giving a reward of +1 only upon full task completion and 0 otherwise. This avoids any complex, task-specific manual shaping and can be applied to any manipulation task.

All models are trained offline using several datasets of trajectories. These offline datasets are collected manually and contain thousands of demonstrations of varying quality. In particular, it includes an extremely small number of expert demonstrations along with a large number of low-quality trajectories generated through random exploration. The model must learn to distinguish distinct quality examples and try to generate the best action sequence.

### 5.3. Baseline and Evaluation Indicators

For this research, the Hierarchical Actor-Critic algorithm is selected as the main baseline for performance comparison. To ensure a fair comparison, the HAC baseline is provided with access to the same library of pre-trained action primitives as used in our hierarchical framework. Specifically, the high-level policy in HAC is allowed to select among the available primitives, matching the action abstraction and low-level control capacity of the proposed method. This design controls the effects of action primitives’ availability and focuses the comparison on the high-level decision-making mechanism.

In addition, to investigate whether the success of DT-HRL is due to all improvements or just 1–2 of them, this paper further conducts a series of ablation studies as follows:

- DT-HRL + Task Embedding + RTG: A Decision Transformer variant conditioned on a task embedding and a return-to-go signal. This structure uses an encoding of the task identity plus the desired return as conditioning input, similar to the original Decision Transformer but adapted for multi-task learning.
- DT-HRL + Task and Goal Embedding: Variant from approach 1, this method conditioned on a goal embedding without original return-to-go prompt. It directly conditions the policy on the explicit goal state or goal representation, relying on goal-conditioned guidance instead of using an expected reward signal.
- DT-HRL + Task and Goal Embedding + Path-Efficiency Loss: Our method which variants from approach 2, trained with a correction for path-efficiency loss (PEL). This variant incorporates the proposed mechanism, which is introduced to compensate for the loss of optimal trajectory guidance caused by the removal of RTG prompts.

For evaluation metrics, this paper uses two primary criteria to quantify performance: success rate and normalized return. The success rate measures the percentage of episodes in which the agent successfully completes the task. The normalized reward is the total reward achieved by the agent, normalized against a baseline, providing a scale-invariant indicator of policy quality. In some of the experiments, the average number of steps required to complete the task is also reported. This efficiency metric reflects how quickly a method achieves the goal on successful trials. All evaluations are conducted under identical conditions for each method, and results are averaged over multiple runs to ensure statistical reliability.

### 5.4. Experimental Results

**Figure 5:**
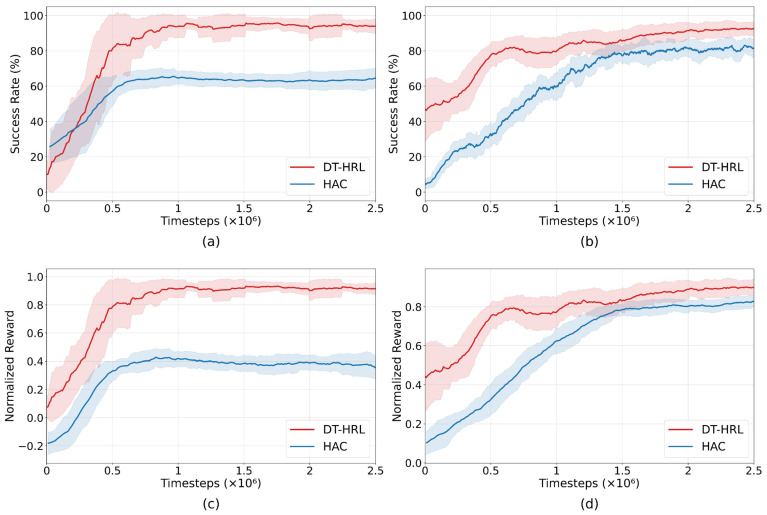
Training curves over HAC and DT-HRL under sparse reward and low-quality datasets. (**a**) Success rate for stacking task. (**b**) Success rate for sorting task. (**c**) Normalized reward for stacking task. (**d**) Normalized reward for sorting task.

The experimental results are summarized in Figure 5. The success rate and normalized reward comparison are shown between the proposed method and the HAC baseline under binary sparse reward settings.

The training curves in Figure 5 present a direct comparison between DT-HRL and HAC baseline on both the stacking and sorting tasks. For visual clarity, curves are uniformly smoothed. The shaded region represents the corresponding moving window range. In both environments, DT-HRL achieves a higher success rate and greater normalized reward than HAC throughout the entire training process.

On the stacking task, DT-HRL demonstrates a rapid increase in both success rate and average reward in the early stages of training, reaching over 90% success rate and a normalized reward close to 0.9. In contrast, HAC converges to a lower plateau, with a maximum success rate of around 65% and normalized reward below 0.5. The gap in learning speed and final performance indicates that DT-HRL not only converges faster but also achieves more reliable and efficient task completion in this long-sequence scenario.

On the sorting task, both methods exhibit gradual improvement, but DT-HRL maintains a clear advantage over HAC. DT-HRL reaches success rates above 90% and normalized rewards near 0.9, while HAC levels off at around 80% for success rate and slightly below 0.8 for normalized reward.

The results show that DT-HRL adapts well to both types of manipulation challenges. Across both tasks and metrics, DT-HRL consistently outperforms HAC, demonstrating better sample efficiency, higher ultimate success, and superior robustness in offline multi-step robotic manipulation.

**Figure 6:**
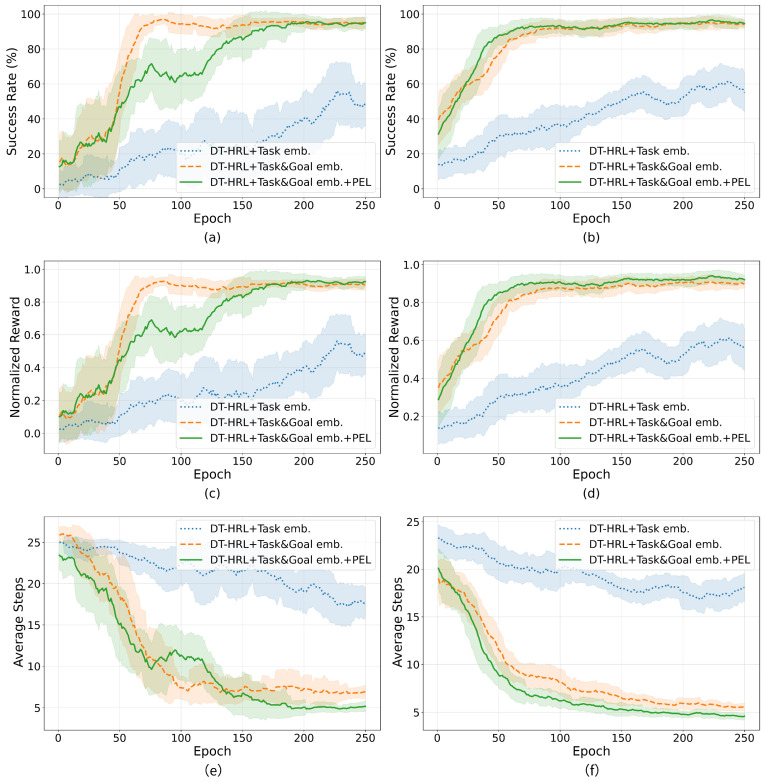
Ablation experiments over 3 variants. (**a**) Success rate for stacking task. (**b**) Success rate for sorting task. (**c**) Normalized reward for stacking task. (**d**) Normalized reward for sorting task. (**e**) Average steps for stacking task. (**f**) Average steps for sorting task.

Ablation Studies:

This paper further evaluates the impact of different components in DT-HRL framework through ablation experiments, as shown in Figure 6. Three variants are compared:

(1) DT-HRL with Task Embedding and RTG;
(2) DT-HRL with Task and Goal Embedding (without RTG);
(3) DT-HRL with Task and Goal Embedding and PEL.

Figure 6 presents the training results of ablation experiments comparing three variants of the DT-HRL framework. The first variant (DT-HRL + Task emb.) uses only task embedding as the conditioning input. The second variant (DT-HRL + Task&Goal emb.) augments the model with explicit goal embedding. The third variant (DT-HRL + Task&Goal emb. + PEL) further incorporates the proposed PEL.

The (1) DT-HRL + Task Embedding + RTG variant achieves a final success rate of approximately 50% on stacking and sorting tasks, with corresponding normalized rewards of 0.5. The (2) DT-HRL + Task and Goal Embedding variant further improves performance, achieving a success rate of about 95% on stacking and 93% on sorting, with normalized rewards rising to 0.9 and 0.87, respectively. The (3) DT-HRL + Task and Goal Embedding + PEL variant leads to the best results, achieving a success rate of nearly 95% for stacking and 94% for sorting, and normalized rewards of 0.92 and 0.93. On both the stacking and sorting tasks, adding goal embedding leads to a clear improvement in both success rate and reward.

While the introduction of PEL does not further increase the task success rate markedly, it significantly reduces the number of steps required to complete the task. This improvement is directly reflected in Figure 6e,f. The (1) DT-HRL + Task emb. variant requires 15–20 steps on average, while adding goal embedding brings this down to approximately 7 steps. With PEL, the average steps further decrease to around 5 steps. This reduction in episode length reflects fewer unnecessary steps and more efficient task execution.

This paper normalizes the score by combining success rate, reward, and average number of steps. To ensure statistical reliability, all results are averaged over 40 independent test runs with different random seeds. Table 3 quantifies the performance differences among the DT-HRL variants using normalized scores, where the reported values are presented as mean ± standard deviation across these runs. The variant (1) achieves normalized scores of approximately 50 and 56 for stacking and sorting tasks, respectively. The variant (2) introduces explicit goal embedding, which significantly improves performance, elevating normalized scores to 92 and 89. The variant (3) incorporating the PEL achieves further improvements, resulting in normalized scores of 94 for stacking and 92 for sorting. These results clearly indicate that explicit goal conditioning provides substantial gains in task performance, while the introduction of the path-efficiency objective further optimizes the quality and efficiency of learned trajectories.

## 6. Conclusions

This paper presented a hierarchical reinforcement learning framework for long-sequence robotic manipulation, drawing inspiration from the layered structure of human motor control. The proposed DT-HRL approach integrates a goal-conditioned Decision Transformer as the high-level policy and a parameterized library of action primitives at the low level. This design enables the system to capture long-term temporal dependencies, generalize across multiple tasks, and efficiently switch between goals in multi-step scenarios.

The proposed framework introduces task and goal conditional inputs to the high-level model, allowing a single network to handle a wide range of manipulation tasks and target configurations. Furthermore, this paper augments the learning objective with a path-efficiency regularization term to encourage direct and efficient trajectories, compensating for the absence of explicit return-to-go signals. The low-level policy, based on a library of reusable action primitives, further enhances learning efficiency and supports transferability to new tasks.

Extensive experiments were conducted on sequential stacking and spatial sorting tasks in simulated warehouse environments. The results show that our method achieves faster convergence, higher final success rates, and greater robustness than the baseline HAC algorithm, especially under sparse reward conditions and with limited expert demonstrations. Ablation studies demonstrate that both goal conditioning and path-efficiency loss make significant contributions to the overall performance.

In conclusion, this study explicitly addresses the current research gaps in long-sequence manipulation. First, by integrating a Decision Transformer with a primitive-based hierarchy, the proposed framework demonstrates improved performance in long-horizon manipulation, thereby alleviating the difficulties that traditional HRL methods face in modeling long-term temporal dependencies. Second, the experiments under sparse reward and low-quality dataset settings show that the framework converges faster and achieves higher success rates than HAC, demonstrating its ability to address a key limitation of existing methods that pure reinforcement learning often requires carefully engineered reward functions, while pure imitation learning relies heavily on expert demonstrations. Third, the evaluation on different tasks with randomized configurations illustrates that the proposed approach overcomes the limited generalization and goal-switching capacity observed in prior methods.

While promising, this study has several limitations. All experiments were conducted in simulation, and real-world deployment may introduce additional challenges such as sensor noise, actuator inaccuracies, and environmental uncertainties. Moreover, the evaluation tasks are restricted to stacking and sorting, which do not fully cover scenarios involving large numbers of objects, dynamic obstacles, or deformable materials.

Future work should focus on validating the proposed framework on physical robotic platforms to assess its robustness under real-world uncertainties. In addition, future research will be extended to a broader set of tasks and more complex environments to provide a richer evaluation of the proposed framework. Moreover, extending the primitive library will be essential for scaling to tasks with higher complexity or requiring precise coordination.

## Figures and Tables

**Table 1:** Comparison of DT-HRL and existing HRL.

Method	HRL	DT-HRL
High-level Policy	Typically a reinforcement learning trained policy network	Improved Decision Transformer
Low-level Policy	Typically a reinforcement learning trained policy network	Reusable action primitives
Sub-goal setting	Often implicit or based on manually designed features	Explicit action primitives with clear semantics
Reward shaping	Often requires carefully designed dense reward	Only requires sparse reward
Generalization	Limited generalization ability without retraining	High generalization ability with general action primitives library
Training method	Requires extensive online interaction	Can be trained offline from low-quality datasets

**Table 2:** Hyperparameters setting.

Parameter	Value
Transformer decoder layers	4
Attention heads	4
Context length H	20
Discount factor γ	0.99
PPO clipping threshold ϵ	0.2
Learning rate η	$1\times 10^{-4}$
Penalty coefficient $\lambda_{\mathrm{PE}}$	0.3
Dropout rate	0.1
Batch size	256
Training epochs	250

**Table 3:** Comparison of normalized scores in distinct tasks.

Task	(1) DT-HRL + Task Embedding + RTG	(2) DT-HRL + Task & Goal Embedding	(3) DT-HRL + Task & Goal Embedding + PEL
Stacking	50±10.2	92±4.3	94±4.1
Sorting	56±8.4	89±5.2	92±3.7

## Data Availability

Data are contained within the article.

## Abbreviations

The following abbreviations are used in this manuscript:

DRL	Deep Reinforcement Learning
HRL	Hierarchical Reinforcement Learning
PPO	Proximal Policy Optimization
HAC	Hierarchical Actor-Critic
A3C	Asynchronous Advantage Actor-Critic
DT	Decision Transformer
SMDP	Semi-Markov Decision Process
DT-HRL	Decision Transformer-based Hierarchical Reinforcement Learning
NLP	Natural Language Processing
RTG	Return-To-Go
PEL	Path-Efficiency Loss
MTGC-DT	Multi-Task Goal-Conditioned Decision Transformer
